# Notes on Some of the Newer Methods of Treatment of Nervous and Mental Disease

**Published:** 1896-06

**Authors:** Frederick Peterson

**Affiliations:** Chief of Clinic, Nervous Department, Vanderbilt Clinic, College of Physicians and Surgeons, New York; Visiting Neurologist to the City Hospital; Consulting Physician to the Manhattan State Hospitals for the Insane


### Texas Medical Journal.

ESTABLISHED JULY, 1885.

Published Monthly.—Subscription $2.00 a Year.

Vol. XI. AUSTIN, JUNE, 1896. No. 12.

### Original Contributions.

For the Texas Medical Journal.

### NOTES ON SOME OF THE NEWER METHODS OF TREATMENT OF NERVOUS AND MENTAL DISEASE.

BY FREDERICK PETERSON, M. D.
Chief of Clinic, Nervous Department, Vanderbilt Clinic, College of Physicians and Surgeons, New York; Visiting Neurologist to the City Hospital; Consulting Physician to the Manhattan State Hospitals for the Insane.

THERE are few conditions in the endless variety of diseases coming under the observation of the medical practitioner so difficult to treat satisfactorily as those which concern the nervous system. Wonderful has been the strides of science in all directions during the past ten years, and that of neurology has kept pace in divers ways with its sister sciences, for have we not unveiled many mysteries in the domain of the brain and spinal chord, disentangled many curious plans of structure, solved many remarkable problems of function and of localization, and brought the pathology of many nervous and mental diseases into the clear light of day? No one gainsays this, and there have been real triumphs of modern medicine resulting from such discoveries, particularly in the field of surgery. There have been revelations, marvels, undreamed of successes in the new surgery of the nervous system. And yet it must be confessed that the new surgery concerns but a restricted class of these disorders, and the great majority of nervous diseases have still to be regarded as obstinate and unyielding, still to be looked upon as unsatisfactory to deal with, still to be treated in a more or less empirical manner. The therapeutics of nervous and mental disease has not kept pace with our great progress in the realms of anatomy, physiology and pathology. Still, advance has been made even in therapeutics, which should be after all the most important part of our aims, for if we are not to prevent and cure the diseases which we so laboriously and successfully investigate, to what end should our researches tend? It is my purpose in this brief paper to touch upon a few methods of treatment, some new and some revivified from an older time, methods with which you are already more or less familiar, but which I wish to amplify and comment upon from the results of my own experience. I shall first speak of several general therapeutic applications and procedures, and then of some particular conditions, diseases and drugs.

### REST.

It was in 1860 that Hilton began his series of lectures on rest and pain, in which he pointed out how much rest had to do with growth and repair of the bodily tissues, and fifteen years later Mitchell wrote of the value of rest in the treatment of hysteria and neurasthenia. Nowadays, however, we apply the principle of rest to a great variety of disorders. Besides its indication in many cases of hysteria and neurasthenia, we find it of the greatest benefit in all sorts of nervous and mental troubles, and especially in such as evince a tendency to waste of tissue and to exhaustion. Most cases of acute mania need to be treated by rest, which should be made as absolute as possible. Many cases of acute melancholia recover more quickly when confined to bed. There are cases of epilepsy in which subjection to rest treatment modifies the attacks in their severity and frequency. Mild types of chorea do not need such aid, but the medium and severe grades of chorea are always distinctly benefitted by more or less protracted rest in bed. While in many nervous and mental cases the rest should be absolute for a period of several weeks in order to insure a successful termination, it is astonishing how much benefit can be obtained by a modified rest treatment, that is, by merely prolonging the daily amount of repose in bed. Early going to bed and late rising are not only good for many an overworked student, housewife or business man, for many a mildly neurasthenic or hysterical patient, for numerous cases of incipient mental disturbance, and for epileptic and choreic patients, but it is frequently used in such disorders as locomotor ataxia, habit spasm, vertigo, anæmia of the brain and cord, exophthalmic goitre, writers' cramp and other professional neuroses (which indeed are local expressions of general nervous exhaustion), hypochondriasis, and kindred conditions. The principle is to apply rest methodically and in proportion to the degree of nervous exhaustion, strain or irritation.

When rest is made nearly absolute, it is necessary that tissue metabolism should be encouraged by attention to the amount and quality of food, and especially by substitution of some passive artificial exercise for the active movements upon which the organism has hitherto depended. This is accomplished chiefly by massage.

### THE ALIPTIC ART.*

Now, massage was a favorite remedy and luxury in ancient Roman times, when it figured as the Aliptic Art, so that it is not at all a new remedy, but its vogue in recent years has assumed enormous proportions, and it has received a scientific study and systematization to which the ancients were strangers. This rubbing, beating and kneading of the trunk and limbs, when skilfully done, is an essential adjunct to the absolute rest treatment. Moreover, it has great importance in all sorts of muscular atrophies. It is invaluable in many kinds of pain, and it often surpasses drugs as a soother of irritation and an inducer of sleep.

I have already alluded to the question of diet.

### DIET IN NERVOUS AND MENTAL DISEASES.

It is needless to say that in connection with a form of rest treatment, simplicity should be the rule as regards food. The selection should be made from the point of view of easy digestibility, and foremost in this regard stand milk and its various preparations. Where milk can not be taken in its ordinary form, some more digestible preparation may be employed, such as peptonized milk, kumyss, matzoon or somal. In cases undergoing a rest treatment this is the main staple of food, and it should be given frequently and in considerable quantity. Overfeeding is indeed another principle in the treatment of any of

---

*The Aliptic Art—A historical study by Dr. Frederick Peterson, Philadelphia Medical News, August 11, 1893.

the nervous and mental diseases in which exhaustion is a feature. Thus absolute rest and over-feeding must be our chief reliance in acute mania, in severe types of melancholia, neurasthenia, hysteria, chorea and the like. Many cases require feeding every hour or two hours. Raw or soft boiled eggs, rare or raw beef, specially prepared cereals and sometimes green vegetables and fruits may be added to the diet. By specially prepared cereals I mean simple boiled rice, stale bread in the form of toast, or better bread which has been twice baked (zwieback.) Stimulants are only occasionally indicated, and then especially in acute maniacal or other dangerously exhausting conditions.

A somewhat similar form of diet is appropriate to neuralgias and mental disturbances having a rheumatic or gouty diathesis as a basis. The same diet is essential in all cases of insanity, neurasthenia, epilepsy and so on, which seem to depend upon auto-intoxication from fermentative or putrefactive changes in the intestinal contents, and such cases we find nowadays to be not at all infrequent.

### HYDROTHERAPY.

The water-cure is not a new thing to your Association, for you have some excellent hydrotherapeutic establishments in Texas. Yet it is only lately that the therapeutic value of water has become generally recognized in this country. When I wrote an article in the February, 1893, number of the *American Journal of the Medical Sciences*, on "Hydrotherapy in the Treatment of Nervous and Mental Diseases," there was really no place in the city of New York to which one could send patients and have his own idea as to treatment faithfully carried out; nor did I know of a single asylum for the insane in this country installed with hydrotherapeutic apparatus, such as I had seen in a number of asylums abroad, even in so remote a country as Greece. Now I could name several public and private asylums which are equipped with arrangements for this purpose. Abroad, this method of treatment has taken a stand second to no other. In the Vienna University there is a chair devoted to this branch of therapeutics, and as regards the particular value of hydriatric procedures in the class of diseases under consideration, the incumbent of that chair, Prof. Winternitz, told me that three-fourths of the patients in his institute for hydrotherapy were sufferers from nervous complaints.

Water affects the nervous system in a variety of ways.

Cold baths increase, and warm baths diminish the irritability of the brain and spinal cord in a reflex manner by stimulating the sensory and vaso-motor nerves of the skin, thus influencing the cerebro-spinal circulation.

Short cold baths, especially when combined with sprinkling, showering, or rubbing, are powerfully stimulating, exhilarating and tonic. Cold baths stimulate peristalsis and the visceral reflexes in the cord, and increase blood-pressure. Prolonged warm baths, steam and hot air baths, and the hot pack, are relaxing and tend to induce sleep. Warm baths diminish arterial tension, and reduce the irritability of individual nerves and the whole nervous system. The spinal douche is of the greatest service in many nervous disorders, because of its remarkable tonic, revulsive and derivative effects. It is a powerful mental as well as physical stimulus. By means of various nozzles it is ejected in the form of a strong stream up and down the back of the patient for a few seconds only, at a distance of some ten feet. Patients with good reaction do not need any special preparation, but at the beginning it is well to have the patient take a warm bath or stay a few minutes in a hot air box previous to its application. At the first seances, the water should not be too cold. Later it may be gradually lowered 50 per cent. Fahr. It should be taken every day when possible. Occasionally this cold spinal douche is alternated with a hot douche (the so-called Scotch douche). This is an exceedingly successful procedure in many cases of hysteria, neurasthensia, and in lethargic and hysterical forms of insanity, where there is sluggish intellect, great depression, apathy, stupor, catalepsy, etc., and in any case of nervous and mental disease where anæmia, chlorosis or gastric trouble exists. I have seen it effective in diminishing some of the symtoms even of locomotor ataxia.

In insomnia there is no other remedy so generally efficient, and at the same time so innocuous. I have seen it successful in wakefulness from every kind of cause, and in cases seemingly intractable to other remedies. Not long ago an especially instructive case presented itself. A distinguished general practitioner in New York brought me a man from the northern part of the State, who had been a victim of insomnia for seventeen years. He had consulted many physicians, and there was no hypnotic drug in the pharmacopœia which had not been tried for long periods. They had of course for a time produced sleep, but only in large doses, and the man was utterly shattered in his nervous system by such treatment. He was tottering and tremulous, his stomach disordered, his mind beginning to be enfeebled, and his sleeplessness only diminished by large doses of deleterious drugs. It was evident to me that hypnotics were no longer to be thought of in such a case. I had him begin the same evening with the hot wet pack. He had a full night's refreshing sleep, and it has since continued to be so successful. The harmful drugs have been banished, and the man is rapidly recovering his almost forgotten health. There are two hydriatric procedures for the production of sleep. One is the prolonged warm whole bath at a temperature of 70% to 90% Fahr., for from one-half to two hours just before retiring. This is indicated in mild cases of insomnia. But the hot wet pack is more effectual, and more widely applicable in all forms of sleeplessness, whether in nervous or insane individuals. It is applied in this way: A blanket 9x9 feet is spread upon the patient's bed, and upon this a sheet wrung out dry after dipping in hot water is laid. The patient lies down upon this; and the sheet is at once evenly arranged about and pressed around the whole body with the exception of the head, after which the blanket is also immediately likewise closely adjusted to every part of the patient's body. Other dry blankets may now be added as seems necessary. The patient remains in this an hour or longer; all night if asleep.

I know of no better treatment of acute maniacal conditions, for instance, than rest in bed, over-feeding and the hot wet pack. In severe forms it is sometimes absolutely necessary to use a drug, and I give sulphate of duboisin hypodermically in 1-100 to 1-60 grain doses, but it is surprising how many cases will do well on this treatment with no drug at all.

A nightly cold foot bath with some chafing of the feet has been exceedingly successful in my hands in the treatment of one of the most obstinate disorders as far as drugs are concerned, viz: congestive headaches.

But the uses of this valuable therapeutic agent are much more diverse than I have space to detail here, aud it is gratifying to observe how widespread the employment of hydrotherapy is becoming from year to year.

ELECTRICITY.

In the domain of electro-therapy, there have been considerable strides of late years. We have attained a greater knowledge of the effects of different currents. For instance, I have experimented with for some years, and described in several papers, that popular effect of the continuous current which goes under the name of cataphoresis, anodal diffusion, electrical osmosis or voltaic narcotism.* I do not know of anything better than cocaine cataphoresis for the immediate relief of trigeminal neuralgia of peripheral origin. To use morphine in such cases is, to my mind, criminal, since the patient is almost certain to acquire a morphine habit. Cocaine cataphoresis gives relief for from five to ten hours. It is also useful in local spasm, such as blephan spasm and facial tic. For general conditions, such as gout, syphilis, and rheumatism, which induce various nervou disorders, the cataphoretic bath can be employed with greater success than any other kind of treatment.

Besides these conditions, it is doubtless through cataphoresis that tropic disorders are influenced, as, for instance, when the galvanic current is given for muscular atrophies. It is the cataphoretic effect of the current that transfers molecules of protoplasm in its path from one cell to another, from the cells into the blood stream, and from the blood stream into the tissues. Hence in muscular atrophies we have this important guiding principle; since the amount of cataphoretic effect depends upon the current strength, we need to employ very large electrodes (covering, for instance, the whole atrophied part), or local immersion of the part in an anodal bath, and to make use of as many milliamperes of current strength as possible.

Another new thing in electro-therapy has been the introduction of the sinusoidal current. This is, in brief, an alternating current, analagous to the secondary current of the Faradaic bat-

---

*Papers by the author: Electrical Cataphoresis as a Therapeutic Measure.—N. Y. Med. Journal, April 27, 1889.

A New Method of Accurate Dosage in the Cataphoretic Use of Electricity.—N. Y. Med. Journal, October 15, 1890.

Further Studies in the Therapeutics of Anodal Diffusion.—N. Y. Med. Record, January 31, 1891.

Introduction of Drugs into the Human Body by Electricity.—Philadelphia Times and Register, March 31, 1891.

Electricity in the Diagnosis of Nervous Diseases.—Buffalo Med. ical Journal, October, 1892.

Chapter on Cataphoresis in Bigelow's International System of Electro-therapeutics. Philadelphia and London: 1894.

Methods of Employing Electricity in Nervous Diseases.—Buffalo Med. Journal, November, 1895.

tery, but with an enormous number of vibrations per second. A great deal has been said, perhaps too much, in favor of this new current, especially in France. Some three years ago Mr. Kennelly and I experimented, at the Edison Laboratory, with the sinusoidal current. The results of these experiments have never been made public, for lack of time, until the present moment. We established one singular and interesting fact, which is of therapeutic value, and which I will detail here. The experiment was tried upon Mr. Kennelly, Dr. Charles E. Atwood, one of the assistants at the Vanderbilt Clinic in the nervous department, who kindly aided us, and upon myself. The same results were obtained in each of us. Applying one pole to a nerve trunk, say at the wrist, and another at an indifferent point, there were no perceptible effects as long as the vibrations were below 2000 per second. When we reached that point the parts supplied by the nerve beneath the pole became anæsthetic, so that pricking with a needle or knife, or touching the part, were not perceived. Both the anæsthesia and analgesia were so marked that an incision might have been made without the consciousness of the individual operated upon. The higher the rate of vibration, the more noteworthy was this effect. Our apparatus did not permit of our going beyond 3000 vibrations per second. We seemed to set up a vibration in the nerve which abrogated the normal nerve vibration, and thus prevented the transmission of sensations to the brain. The return to sensibility was instantaneous on interruption of the electric current. Doubtless small operations might be performed by this new method of local anæsthesia. As yet, the procedure is in its infancy. It will relieve severe neuralgias temporarily, and, I presume, as the method is more perfected it may be productive of gratifying results in this field, much better than by any other system of vibratory application.

The recently discovered X rays of Roentgen will not only bring out remarkable facts in connection with the whole subject of light and electricity, not only have a use in surgical diagnosis, as has been already demonstrated, but are destined, I am sure, to aid us in the investigation of the structure of the central nervous system. We will very likely not be able to photograph the brain, but we can photograph sections of it of varying thickness, and of parts of the spinal cord and nerves, and, taking advantage of the varying conductivity of metals as regards the X rays, and of the susceptibility of different nervous tissues to certain metal stains, we shall probably be able to definitely increase our already large knowledge of nerve and cell relations.

### VIBRATORY THERAPEUTICS.

The employment of vibration in nervous disorders is not exactly new, but it received a new impulse when Charcot and his school, not long ago, reintroduced it in the treatment of certain affections. There is no doubt of its usefulness in a variety of disorders, such as neuralgia, neurasthenia, hysteria, and headaches. A vibrating chair has been found serviceable for cases of paralysis agitans. I have noted marked benefit in obstinate tinnitus aurium from vibration over the temporal bone. The apparatus I use is various. This cap I had made for me by Messrs. Waite and Bartlett, of New York. It consists of parts for adjustment to the head. Upon the vertex a small electric motor is fixed, and by means of a small lever with ball attachment (an eccentric) the vibration may be made slow, coarse, fine or rapid, as one may deem expedient, and the rate of vibration may be determined. Another instrument made for me by the same firm, is a modification of the electric engraving tool, adapted to the administration of vibration to definite points along nerve trunks. Another apparatus that I make use of is one patented and manufactured in Sweden. With this, vibration of any degree may be given to any part of the body, by means of diverse kinds of handles, to the trunk, extremities, head, and even to the larynx and eye. I presume the therapeutic effects of vibration consist of nutritional changes induced by the shaking up of the parts to which it is applied, and of modifications in nerve-impulses from the counter-waves produced by the apparatus.

### AUTO-INTOXICATION.

Researches in the physiological chemistry of digestion, as well as observations in many pathological conditions, have established that auto-intoxication from the absorption of poisonous substances generated in the alimentary canal by putrefactive processes is not only a real thing, but a frequent factor in the etiology of a number of nervous disorders, such as headaches, neurasthenia, hysteria, neuralgia, and even graver maladies, like epilepsy, melancholia, and mania. It behooves us, therefore, in these diseases to investigate carefully for evidence of any such cause. Periodical or constant attacks of gaseous diarrhœa are somewhat indicative of this condition. Frequently the conditions of the bowels furnish no information of the actual state of affairs. Recent researches* tend to show that an excess of etherial sulphates in the urine (indican) is a good index of auto-intoxication in connection with other symptoms.

When auto-intoxication is suspected as the causative factor in any nervous disorder, it is essential to regulate the diet in the manner already mentioned, and there are at our disposition a number of intestinal antiseptics which, though not always efficient, are yet often of very great benefit. I have found in my own practice that beta-naphthol is one of the best intestinal antiseptics. I give it in capsules of 5 grains each, two hours after eating, with water. In several cases of epilepsy and of melancholia it has acted exceedingly well. In several cases of epilepsy, salicylate of soda has also proved itself to be of great value. Salol, too, is a good intestinal antiseptic. Sometimes I have made excellent use of peppermint for the same purpose. I think the abundant use of water a necessary adjunct in the treatment, usually advising the drinking of hot water several times daily on an empty stomach, and sometimes adding thereto frequent flushing of the large intestine with warm water.

### MYXOEDEMA AND EXOPHTHALMIC GOITRE.

One of the remarkable advances of recent years has been in relation to the thyroid gland. I need not dwell too long upon a subject already made familiar by the published observations of many writers. Myxœdoma in the adult, and its corresponding condition in the young, infantile myxœdema, or cretinism, are nutritive disorders depending upon a diminished or absent secretion of thyroid juice. While not strictly nervous diseases, they have interested neurologists, because in the adult this condition leads to mental dullness and insanity, and the growing brain of the infant and child to retardation of mental development and cretinous or myxœdematous idiocy and imbecility. These conditions are no doubt rare in the west, and infrequently met with, but in the great material that is congregated in a large city, it is by no means rare or uncommon. I have observed the results of treatment in some seven cases of myxœdema and in at least a dozen cases of cretinism, and the effects are truly marvellous, as they have been described. I am about to publish the results of thyroid treatment in two or three cretins that have

* Herter and Smith, New York Medical Journal.

been under my observation as neurologist to the Randalls' Island Hospital for Idiots.

Now exophthalmic goitre, or Graves' or Basedow's disease, as you may please to call it, is a malady in which the pathology has been, but is growing less, obscure. Until recently we were content to look upon it as a nervous disease. Nine years ago, in a careful study of this subject,* I wrote:

"In my opinion an anatomical lesion in the cardio-inhibitory nerve-path, or its medullary centre, which diminishes but does not destroy its functional activity, is the cause of Basedow's disease."

But in view of the discovery of the function of the thyroid gland, which secretes a substance necessary to the proper regulation of metabolism, I am prepared to alter that opinion. There seems, nowadays, to be considerable justification for the theory that exophthalmic goitre is due to either an increased or a perverted secretion of thyroid juice, and probably the former. This seems to be borne out by the strong contrast existing between the symptoms of myxoedema and Graves' disease, by the effects of overdosing in treatment with the thyroid extract, and by the frequently favorable results of compressing the thyroid gland or exsecting portions of it in Graves' disease. On this new theory, and it is more than probable that this is the correct one, the principle of treatment in exophthalmic goitre is to diminish, as far as possible, the amount of thyroid secretion. The exsection of a part of the gland (thyroidectomny) is a medical procedure which is certainly indicated in many cases unless they have already gone too far. It has cured many cases of Graves' disease. Compression of the gland by bandages, or by a special apparatus, which I have had made, and which has acted well in some instances, has for its object the same idea. Ligature of one or more vessels supplying the gland may be tried, and I have the conviction that galvano-puncture will one day prove a very efficient means to the same end. It is possible that drugs which diminish secretion (like belladonna) may be useful in certain cases. Perhaps an agent may be discovered ere long which will neutralize the effects of the excess of thyroid secretion in the system, and this is a problem for the physiological chemists.

The remarkable effect of thyroid extract upon general nutri-

---

* Morbus Basedowii. By Frederick Peterson, M. D., N. Y. Medical Record, August 20, 1887.

tion naturally leads one to consider its possibly useful employment in some of the disorders which we are accustomed to regard as due to nutritional abnormalities in the nervous system, such, for instance, as epilepsy, paralysis agitans, and some forms of insanity. I have been treating, for some time, ten selected cases of epilepsy, and some cases of paralysis agitans, with thyroid extract, but it is as yet too early to draw any inferences from the treatment.

### TETRA-ETHYL-AMMONIUM.

There are some nervous and mental disorders, such as neuralgia, sciatica, parasthesia, neuritis, local paralyses and occasional psychoses, which depend for their origin upon the rheumatic or gouty diathesis. Where such seems to be the causative factor, I prescribe tetra-ethyl-ammonium in addition to any other particular remedy indicated. I introduced this drug to the medical profession in an article upon it in the *New York Medical Journal* for September 16, 1893. Its extraordinary efficiency as a solvent for uric acid was discovered by Edison, the inventor, at his laboratory shortly before, and given by him to me for investigation as to its medicinal virtues. It is given in doses of 10 to 20 drops of a 10 per cent. solution three times daily, well diluted to begin with, gradually increasing the dose if necessary. An interesting point in connection with this drug is that it exists normally in the organism, and it is theoretically possible that a want of it may be responsible for many of the manifestations of both gout and rheumatism. At any rate, treatment with it has been, in my hands, very gratifying in many instances, and it deserves more attention than has as yet been given it.

### CORD-STRETCHIHG IN LOCOMOTOR ATAXIA.

Suspension by means of the Sayre apparatus is a means of treatment in tabes that swept over this country several years ago, and which has fallen almost wholly into disuse; first, because it did not give all of the results it seemed to promise, and secondly, because of the trouble and even danger (syncope occasionally) involved in its employment. But doubtless the method had in it an element of usefulness, and the very slight stretching of the spinal cord incident thereto was, in a few instances, productive of good. Whatever of benefit was derived from suspension may be obtained by a much more simple, and at the same time much more efficacious method of stretching the spinal cord, viz: the Bonuzzi method, which I recommend to every case of locomotor ataxia in my own practice, but which I believe is not familiar, if it be known at all to neurologists or general practitioners in this country. The procedure is briefly thus: the patient lies upon his back on a sofa. The operator lifts up both of his legs and flexes the thighs as far as he possibly can, the patient keeping his knees perfectly extended. The toes approach toward the head, and in this way the back becomes very much arched, and experiments upon the cadaver have demonstrated an actual and considerable stretching of the spinal cord. At first, the uncomfortable position is to be maintained for but a few seconds, but the daily or tri-weekly seances may be gradually prolonged to but a few minutes or more. I have seen under this treatment some of the troublesome symptoms of tabes disappear, such as the ataxia, pains, and disorders of the sphincters.

### TREATMENT OF ALCOHOLISM AND ALCOHOL INEBRIETY.*

The treatment of alcoholic conditions may be divided into three categories, in all of which nitrate of strychnia, originally introduced by a Russian physician for this purpose, given hypodermatically, I find to be the most efficient agent. These three categories are acute alcoholism, chronic alcoholism, or alcoholic neurasthenia, and the alcohol habit.

#### ACUTE ALCOHOLISM.

1. Cut off all alcohol, and confine to bed.
2. Blue pill at night, followed by saline cathartic.
3. Hot wet pack for sleeplessness.
4. Hypodermatic injection of nitrate of strychnia, gr. 1-60 to 1-32.
5. Water, milk, kumyss, broths, soup, meat juice, raw eggs, arrowroot, juicy fruits and the like, when there is gastric disturbance.

This is the outline, in short, of a kind of treatment adapted to all cases of acute alcoholism, though bromide and chloral, or duboisine, are indicated in a certain number of instances.

#### ALCOHOLIC NEURASTHENIA.

1. Cut off alcohol.
2. Hot wet pack for insomnia.
3. Disturbances of the alimentary canal to be met by ape-

---

*The Treatment of Alcoholic Inebriety. By Frederick Peterson, M. D., Jour. Am. Med. Assn., April 15, 1893.

rients and dyspepsic remedies (rhubarb and soda, hydrochloric acid and the like). The diet should be milk, eggs and vegetable foods, meats rarely.

4. Strychnia again the main agent to restore nerve tone; best given hypodermatically, but may be given by mouth in combination with quinine, or in fluid extract of cinchona (1-60 to dr. j.), or in infusion of gentian.

As regards the alcohol habit or alcohol inebriety, the Keeley cure, while it has made use of no drug not long ago tried by physicians all over the world, served, at any rate, to bring before the profession the great value of repeated suggestions in the treatment of this class of cases. Most of us have been accustomed to pay too little attention to these cases, and to dismiss them with a prescription and friendly advice. I wish, therefore, to emphasize the particular advantage of having the inebriate patient come twice daily for a hypodermatic injection of strychnia. It is the continuous attention and suggestion of these daily visits which avail in the disorder, the strychnia of course acting as a prop to his nervous system deprived of its habitual stimulant. I would outline thus, then, the treatment of

THE ALCOHOLIC HABIT.

1. The hypodermatic injection of nitrate of strychnia in the doses already given, at least twice daily, more frequently, if possible, and always by the physician himself. The moral influence and personality of the physician are of greatest importance. By this frequent contact of physician and patient the effort and attention of the inebriate are kept continually at their highest pitch.

2. A diet of milk, eggs and vegetable foods should be enforced, meats being allowed but once daily.

3. Regular occupation, regular hours, and the avoidance of the society of fast companions should be insisted upon.

4. There is a certain class of patients to whom a substitute for a dram of liquor is at times imperative; when the desire comes on it must be satisfied. The substitute must be immediately at hand. With some of these, a combination of strychnia and fluid extract of cinchona (gr. 1-60 to dr. j) taken with a glass of water, works very well. It is not always convenient, however, to carry a bottle in the pocket, so I am at times in the habit of prescribing powders composed of from twenty to forty grains of red cinchona bark, half a grain of capsicum, and three grains of powdered nux vomica, to be taken with a glass of water when required.

### NEW THERAPY IN EPILEPSY.*

Any remedy that offers any sort of success, even in a limited number of cases of epilepsy, is more than welcome. This is one of the most common of nervous disorders, two in a thousand of population being afflicted, and it is also one of the maladies with which the profession have been well acquainted clinically for two or three thousand years. It might also be called the *opprobrium neurologicum* from the fact that so little has been accomplished during this long period, either in regard to its pathology or its cure. We may say, however, that we have recently become more than ever convinced of its manifold pathology. We have come to look upon it more than ever as a symptom of a great variety of pathological conditions. More and more every year do we restrict the number of cases that may be called truly idiopathic epilepsy. We need to examine our cases with the greatest possible care, in order to exclude conditions which may require some particular treatment, such as trauma, tumor, old meningeal hemorrhage; reflex convulsions from genital, nasal, dental, ocular, or gastro-intestinal irritation, or from old cicatrices; epilepsy due to auto-toxæmia and other toxic blood states. But as in most cases, we will find, after the most searching investigation, no cause whatever for the attacks, we are constrained to creat such empirically, and it is to several new empirical methods of treatment that I wish to direct your attention. We will suppose that the bromides, and borax, and belladonna, and the whole category of old remedies have been tried in vain. I will say that solanum crolinensis, or horse-nettle, recently introduced, has had no effect whatever in my cases. On behalf of tincture of simulo, a South American plant of the hyssop family, I can say from an experience of several years, that it is perfectly harmless, and that in several instances it has had remarkably good effect where other remedies have failed. The so-called opium-bromide treatment of Flechsig of great use to many patients, particularly in old and obstinate cases where all other agents have been inefficacious. This treatment consists of the administration of opium for some six weeks, beginning with one-half to one grain three times

---

*The Treatment of Epilepsy—By Frederick Peterson, M. D., American Medico-Surgical Bulletin, February 1, 1895.

daily and gradually increasing until ten to fifteen grains are taken. Then the opium is suddenly stopped, and bromides in large (thirty grains four times daily) and gradually reduced doses are given.

Another new combination with the bromides has been suggested by Bechterew, viz: that of adonis vernalis. As you know, adonis vernalis has much in cemmon with digitalis, which has been used in past years in epilepsy, but the employment of the former in epilepsy and conjointly with the bromides is new, and in several of my cases the result has been more than usually gratifying.

I can not forbear referring here for a moment, in closing, to the moral treatment of epilepsy, which is certainly new, and which has not received, until lately, any of the consideration which it merited. It is only too well known to all of us how epileptics have been dismissed with a prescription and possibly some advice as to regulating their diet, rest and exercise. But the especial needs of this peculiarly unfortunate class of dependents had never been brought fully before the profession. No hospitals receive them. The schools can not take them. No one wishes to employ them. They are ostracised from society, forbidden to take part in the recreations of their fellows, and shunned more or less by everybody. Untaught, idle, sick, neglected, they drift finally into the only shelter offered them—almshouses and insane asylums. But a large majority of them, were it not for their attacks, could be educated in schools, could acquire trades, could enjoy recreations and take a part in the affairs of mankind. Thus it is that a scheme of colonizing them has been undertaken in several of the United States, following the example of Germany and France. I will only allude briefly to the plan already in operation in the State of New York at Craig Colony.* The State has here a tract of nearly 1000 acres of the best kind of agricultural land with already some thirty to forty buildings upon it. Here the epileptics of the State already upon public charge are being congregated

*Papers by the author: The Bidefeld Epileptic Colony. N. Y. Med. Record, April 13, 1887.

Colonization of Epileptics. Journal of Nerv. and Mental Diseases, December, 1889.

Plan for an Epileptic Colony, N. Y. Med. Journal, July 23, 1892.

Care of Epileptics. Journ. Am. Med. Assn., Sept. 30, 1893.

Craig Colony. Pedriatrics. Feb. 16, 1896.

(there are over 1000 to be cared for in this manner) and are to be given education in the usual branches of learning, taught every kind of industrial occupation, to be treated for their malady, and be afforded a home in a sort of village life where they will no longer feel their social isolation nor be debarred from the innumerable privileges enjoyed by the rest of humanity. This moral treatment of epilepsy is by far the greatest stride in advance taken for centuries in the therapeutics of one of the most distressing of nervous diseases.

60 West 50th St., New York.

